# The digital scent device 20: an automated, self-administered odor identification test

**DOI:** 10.1007/s00405-024-08887-4

**Published:** 2024-08-29

**Authors:** Agnieszka Sabiniewicz, Sophia Wittig, Antje Haehner, Christopher Müller, Claudia Galvao, Marco Nakanishi, Thomas Hummel

**Affiliations:** 1 Smell and Taste Clinic, Department of Otorhinolaryngology, TU Dresden, Dresden, Germany; 2 NOAR, Sao Paulo, Brazil; 3 Department of Otorhinolaryngology, University Hospital of Brasília UnB-EBSERH, University of Brasilia, Brasilia, Federal District, Brazil

**Keywords:** Digital Scent Device 20, Olfaction, Odor identification test, Sniffin’ sticks

## Abstract

**Purpose:**

Assessing olfactory function is highly significant in clinical practice, particularly in the context of the recent COVID-19 pandemic. Recent approaches in this field emphasize the importance of reducing the time and cost devoted to olfactory testing procedures. Hence, the aim of the present study was to examine the reliability and basic characteristics of Digital Scent Device 20 (DSD-20), an innovative olfactory test consisting of 20 “universal odors”, in a European population.

**Methods:**

A total of 88 participants (mean age = 45.1, SD = 20.3) volunteered for the study. The sample consisted of 37 normosmic controls and 51 dysosmic patients.

**Results:**

The correlation between DSD-20 and the total score in Sniffin’ Sticks was high (TDI; *R* = .80, *p* < .001), and the test correlated with the individual components of the Sniffin’ Sticks test. Furthermore, the correlation coefficient between DSD-20 test and retest was very high (*R* = .88, *p* < .001), which was additionally confirmed by a Bland-Altman plot. Essential characteristics of the DSD-20 are its simplicity in self-administration, speed of application, portability, and the fact that it can be reused.

**Conclusion:**

Overall, the present study confirms previous notions on DSD-20 by demonstrating its high reliability and usefulness in separating patients with hyposmia/anosmia and normosmic controls.

## Introduction

Assessing olfactory function is highly significant in clinical practice, particularly in the context of the recent COVID-19 pandemic [1]. Olfactory impairments are not only signs of various viral diseases, including SARS-CoV-2 infection [2, 3] but also are a first marker of neurodegenerative diseases, including Parkinson’s disease [4], Alzheimer’s disease [5] or dementia [6]. Furthermore, a link between impaired sense of smell and cognitive and affective decline, as well as impaired quality of life, has been well-established [7–9].

A number of reliable and validated psychophysical tests for olfactory assessment have been developed for clinical practice. Some of them were meant to address the need to validate olfactory abilities in specified populations (e.g., Italian Olfactory Identification Test [10] Scandinavian Odor Identification test [11]), while others, on the contrary, measured the sense of smell among different cultures (e.g., Cross-Cultural Smell Identification Test [12]). The last two decades have witnessed the development of numerous olfactory tests that employed different methods of measuring olfaction, namely Barcellona Smell Test – 24 [13], consisting of both odors affecting the olfactory nerve and those perceived by trigeminal nerve; European Test of Olfactory Capabilities [14] based on odor identification and suprathreshold or self-administered olfactory test for the remote evaluation [15]. Among these, the most widespread are the University of Pennsylvania Smell Identification Test [UPSIT; 16], which employs microencapsulation of odorous liquids, and the Sniffin’ Sticks test [17, 18], which uses pen-like odor dispensers. These objective methods not only measure olfactory performance but also detect cognitive and affective changes [19, 20].

Among all olfactory subtests, the identification test procedure is the fastest and easiest to follow for both patients and experimenters [17, 21]. Identification scores provide information not only about potential olfactory dysfunctions [22] but also about the cognitive performance of participants [23] and depression severity [24, 25]. Furthermore, odor identification impairment is a biomarker for COVID-19 [26].

Recent approaches in clinical practice emphasize the importance of reducing the time devoted to olfactory testing procedures [22]. Such abbreviated versions seem to be a reasonable solution in laboratory or clinical settings since running olfactory tests typically requires at least 10 min to complete. Furthermore, the lack of trained medical personnel has heightened the need for self-administered tests [27]. Instead of dedicating one medical researcher to conduct and score an olfactory test, a patient can conduct self-administered tests on her own. Rather than requiring a medical researcher to conduct and score these tests, patients can administer them independently. All these became particularly essential in the context of the recent COVID-19 pandemic, where fast and self-administered screening simply enabled the examination of more patients and lowered the risk of contagion [15]. Lastly, since olfactory tests, especially those designed for one-time use, are costly, there is a demand for more affordable methods.

Digital Scent Device (Multiscent-20; DSD-20; Noar, São Paulo, Brazil), based on the original Multiscent-40 by Nakanishi and colleagues [28], addresses several challenges in olfactory testing. This innovative olfactory test consists of 20 “universal odors” using a four-alternative forced-choice (4-AFC) paradigm. A recent study on the Brazilian population [29] confirmed its reliability and accuracy; however, no study so far has validated this test in Europe. Hence, the aim of the present study was to examine the reliability and basic characteristics of DSD-20 in a European population in comparison to extensive standardized psychophysical examination.

## Materials and methods

### Participants

A total of 88 German-speaking participants, comprising 42 women and 46 men aged between 18 and 82 years (mean age = 45.1, SD = 20.3), participated in the study. They were recruited either from the Smell and Taste Outpatient Clinic of the Department of Otorhinolaryngology at the TU Dresden or through word-of-mouth or flyers put up at the University campus. Detailed characteristics of the participants are presented in Table 1. None of the participants smoked. The sample size was determined by utilizing G*power software [30]. T-test for two groups, with a level of significance set to α = 0.05 (described in detail in the “Data analysis” section) to detect large effects of d = 0.9 (critical t = 2), the projected sample size was at least 70.

The total sample consisted of healthy controls and a slightly older patient group (t[84] = 4.7, *p* < .001, Cohen’s d = 0.3). Both groups did not differ in terms of gender distribution (χ^2^ = 0.08, *p* = .78). Healthy controls reported significantly better olfactory function compared to patients (t[30.5] = 12, *p* < .001, Cohen’s d = 3.5). Among patients, common causes of olfactory dysfunction were SARS-Cov-2 infection, head trauma, chronic rhinosinusitis with/without nasal polyps, and infections other than SARS-Cov-2. Inclusion criteria were: 18 years of age and subjective smell loss.

The study was performed according to the principles of the Declaration of Helsinki on biomedical research involving human subjects. The research protocol has been approved by the Ethics Review Board at the Medical Faculty of the TU Dresden [BO-EK-556122022]. All patients provided written informed consent.

### Olfactory test: the DSD-20

**Fig. 1:**
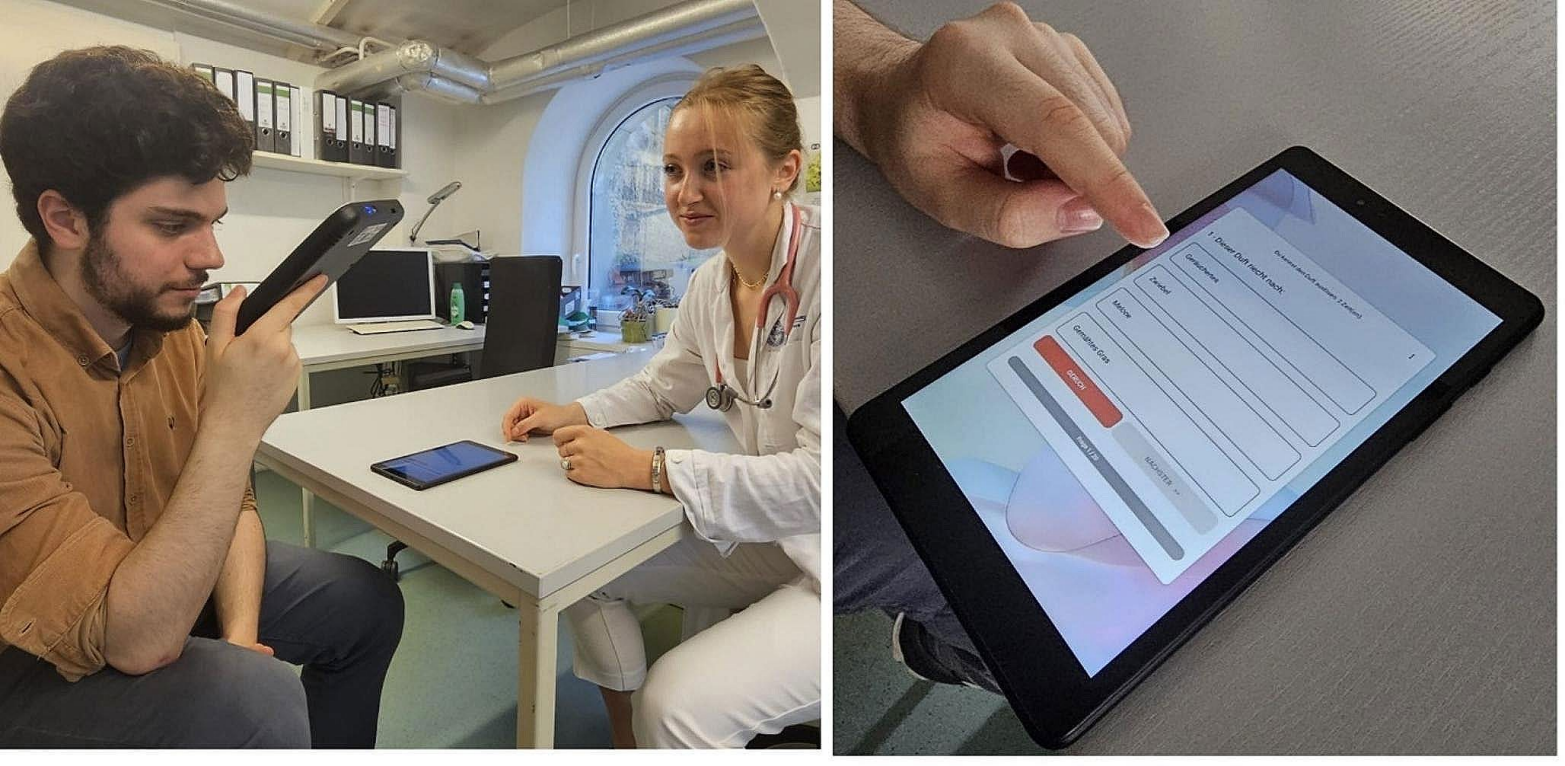
Performance of olfactory testing via DSD-20

The DSD-20 is presented in Fig. 1 Its detailed characteristics have been previously outlined by Nakanishi and colleagues [22]. In summary, the DSD-20 is a tablet-like device designed to present odors. Its odor delivery system consists of 20 micro-cartridges, an air filter, a mechanism that produces a dry air stream, and an odor dispensing opening. The odors are stored in capsules made from an oil-resistant polymer.

Individual odors are released through a small opening at the upper front of the device. The device pumps a small amount of room air through the odor capsules (16 ml/s), leaving little or no residue in the environment or on users. In this study, each odor was presented for 5 s, with a minimum interval of 6 s between repeated presentations of the same odor and a minimum interval of approximately 20 s between presentations of different odors. Each capsule was loaded with 35 µL of oil-based perfume solution, enabling the device to maintain consistent odor intensity and identifiability for up to 100 activations. The odors used in the capsules were complex mixtures prepared by the Givaudan Flavors and Fragrance Corporation (São Paulo, Brazil).

### Procedure and testing

Detailed results of all tests for both healthy controls and patients are presented in Table 1. All participants underwent examinations twice, with each session lasting approximately 40 minutes and conducted on separate days. The assessments included detailed psychophysical olfactory testing using two methods: 1) the DSD-20, and 2) the Sniffin’ Sticks battery. The latter is widely used in both daily clinical practice and scientific research and includes three tests that measure distinct aspects of olfactory perception: olfactory threshold, odor discrimination, and odor identification [17, 18].

The scores from the threshold, discrimination, and identification tests are aggregated to compute an overall TDI score, which ranges from 1 to 48.

### Data analyses

The normality of the data distribution in the DSD-20 test was investigated via the Shapiro-Wilk test. Given the non-parametric nature of the distribution, respectively non-parametric tests were employed in further analyses. Specifically, Welch t-test was employed to investigate the difference in test performance (DSD-20) between the group of patients and healthy controls. Furthermore, to measure DSD-20’s reliability, Spearman correlation was conducted between DSD-20 and, respectively, total Sniffin’ Sticks performance (TDI), as well as odor threshold (T), discrimination (D), and identification (I); DSD-20 test-retest. Additionally, Bland-Altman plot [24] was produced to examine the test-retest reliability of the DSD20 in more detail. Furthermore, Spearman correlation was also employed to obtain demographic characteristics such as the relationship between DSD-20 performance and age in the group of healthy controls. Lastly, among healthy controls, we used Wilcoxon-signed rank to compare performance in DSD-20 test between genders.

## Results

### Descriptive statistics

The Shapiro-Wolf test indicated a non-normal distribution of DSD-20 test scores (Shapiro-Wolf = 0.9, *p* < .001, Median = 12, IQR = 7) with the majority of responses skewing to the right (Skewness = -0.6, SES = 0.3; Kurtosis = − 0.9, SEK = 0.5).

**Table 1:** Characteristics of participants and results for olfactory tests (median ± IQR) separately for the two sessions

Group					
	Healthy controls		Patients		
Number of participants	37		51		
Women	54%		51%		
Age (in years)	24 ± 23		58 ± 29		
Etiology of hyposmia/anosmia (valid percent)			Covid infection (45%), head trauma (23%), other infection (7%), tooth removal (3%), medicines (3%), nasal polyps (3%), unidentified (16%)		
Appointment	Session 1	Session 2		Session 1	Session 2
*Olfactory tests*					
Odor threshold	10.3 ± 4.3	11 ± 3.3		1 ± 3.4	1.3 ± 4.6
Odor discrimination	13 ± 2	13 ± 1		8 ± 5.8	6 ± 6.5
Odor identification	13 ± 2	14 ± 2		8 ± 4.5	9 ± 6
TDI	37.6 ± 6.3	38 ± 4		19 ± 12.5	17 ± 18.5
DSD-20	14 ± 2.8	14 ± 2		8 ± 7	8 ± 6

### Difference between patients and healthy controls

**Fig. 2:**
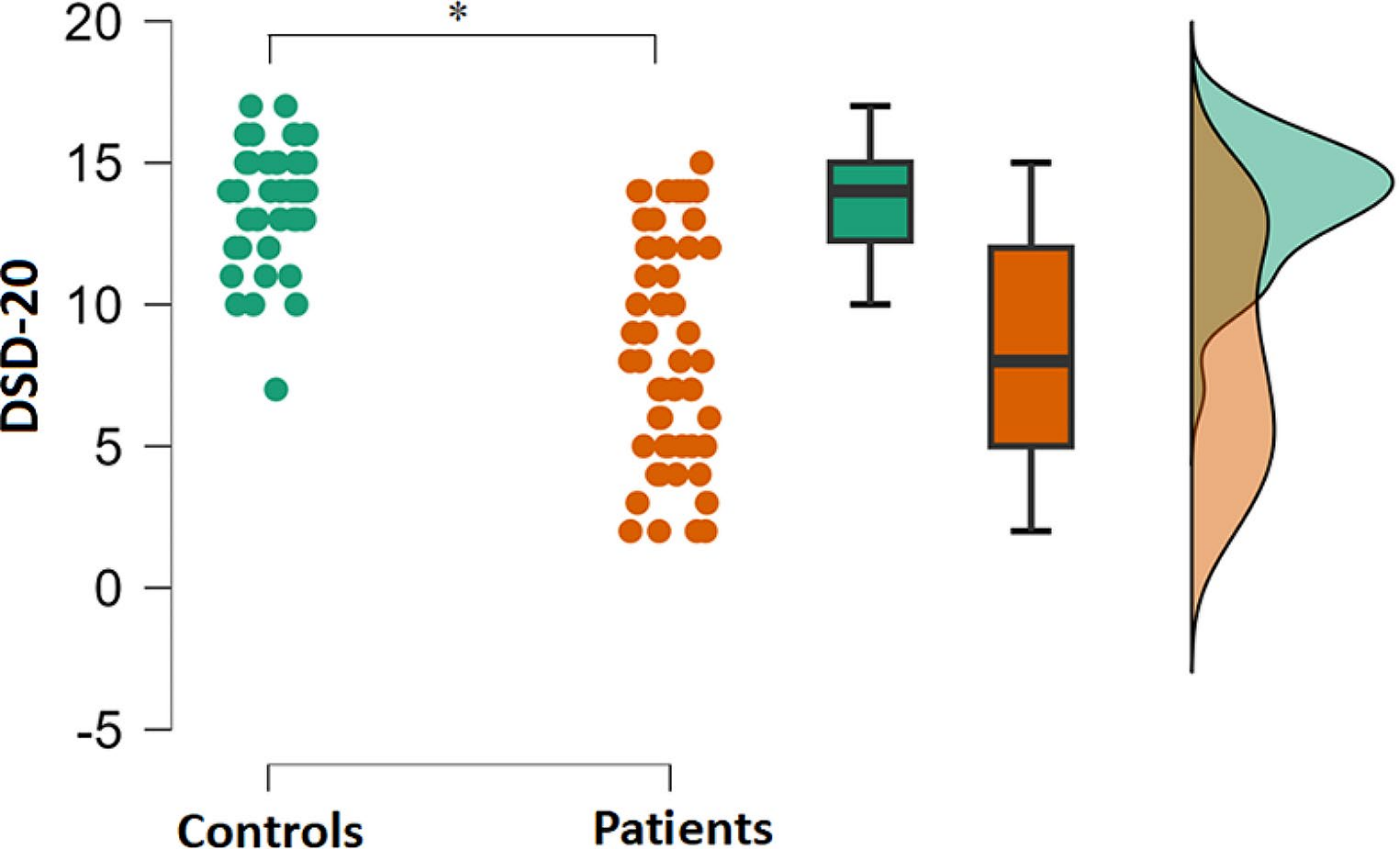
Difference in the DSD-20 performance between the group of patients and healthy controls. Green symbols/areas indicate the results of healthy controls, and yellow symbols/areas indicate the results of patients. Asterisk indicates a significant difference between the groups

Healthy controls scored significantly higher in DSD-20 compared to patients (t[80.1] = 7.6, *p* < .001, Cohen’s d = 1.6; Fig. 2).

## Correlations between DSD-20 first and second session and DSD-20 and Sniffin’ sticks

**Fig. 3:**
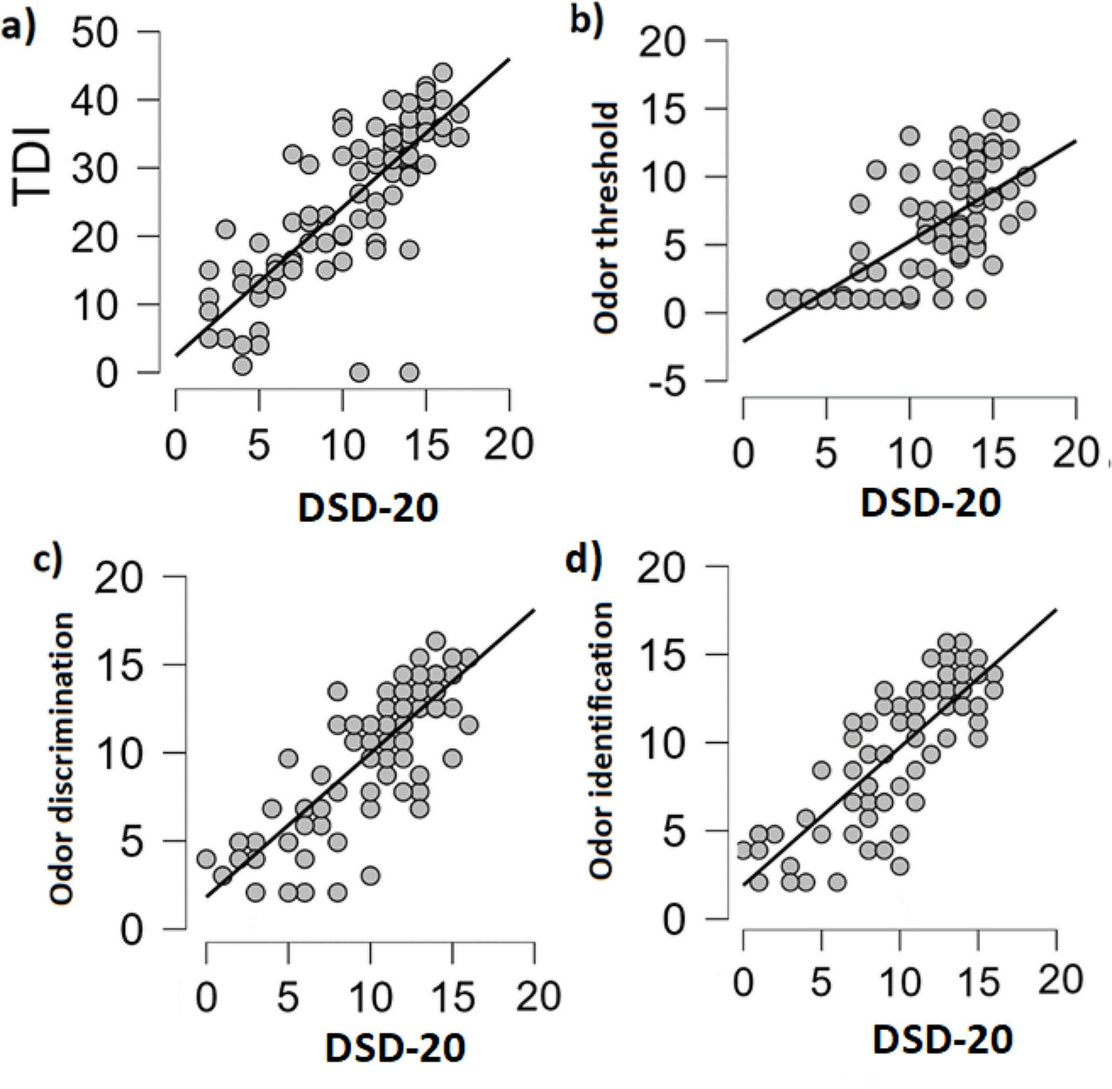
Spearman correlation between DSD-20 and, respectively, (**a**) TDI; (**b**) odor threshold; (**c**) odor discrimination; (**d**) odor identification

DSD-20 correlated highly with total Sniffin’ Sticks performance (TDI) (*R* = .8, *p* < .001; Fig. 3a), as well as Sniffin’ Sticks subtests, namely odor threshold (*R* = .71, *p* < .001), discrimination (*R* = .73, *p* < .001), and identification (*R* = .64, *p* < .001) (Fig. 3b, c,d).

DSD-20 Test-retest reliability was high (*R* = .88, *p* < .001).

**Fig. 4:**
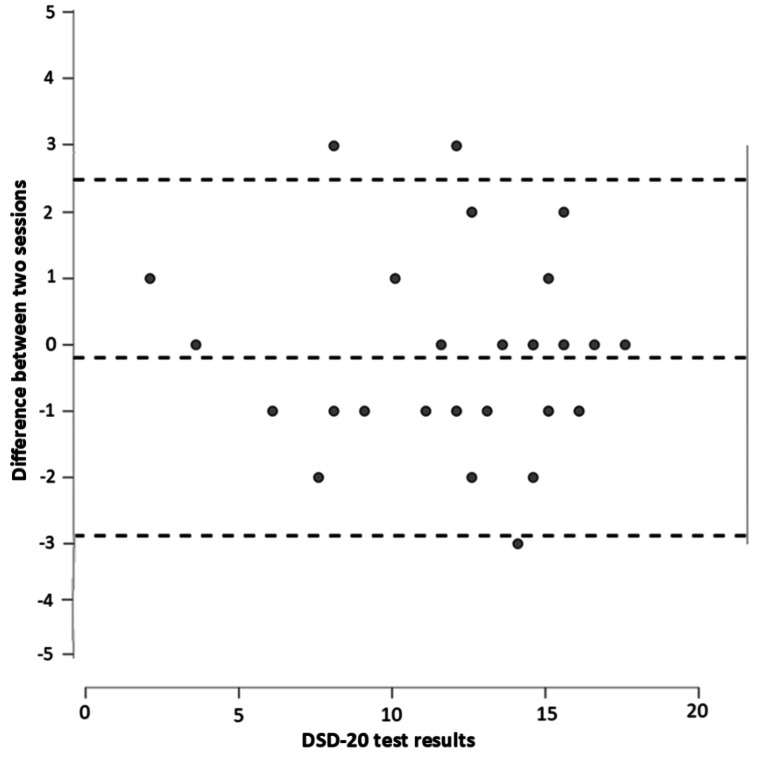
Bland-Altman plot for DSD-20 test-retest. The central dashed line presents the mean difference between the test and retest of -0.2, the upper dashed line stands for the upper boundary of 2.5, and the bottom dashed line identifies the lower boundary of -2.9

Bland-Altman plot (Fig. 4) showed that only 3 of 26 individuals had scores very closely outside of the boundaries of the Bland-Altman plot defined by the difference of the results from the test and retest versus the mean of the two data points. This suggested a good reproducibility of the test results, especially when considering that the three deviant scores were only present for the higher range of scores.

### Demographic characteristics

**Fig. 5:**
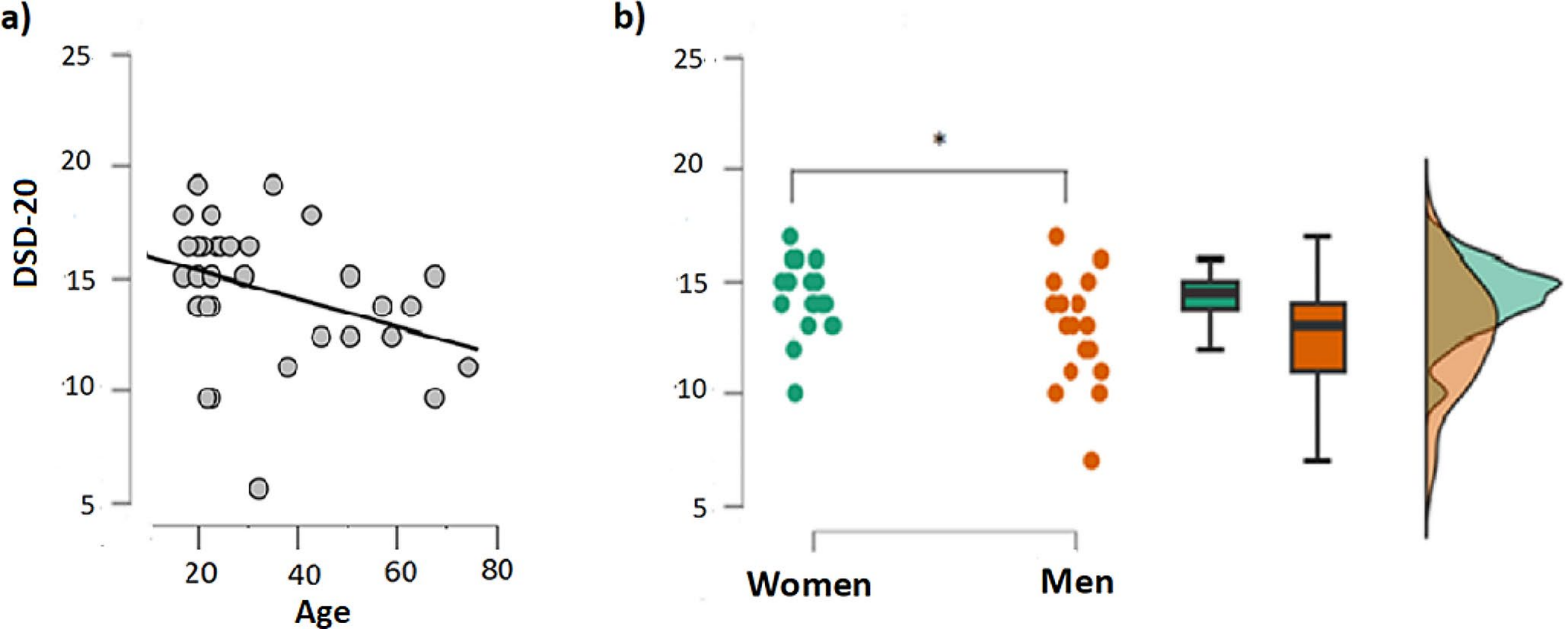
**a)** Spearman correlation between age and DSD-20 performance in the control group; **b)** differences in DSD-20 performance between men and women in the control group, as indicated by t-test. Green symbols/areas indicate the results of women, and yellow symbols/areas indicate the results of men. Asterisk indicates a significant difference between these groups

In the control group, age was negatively and significantly associated with DSD-20 performance (*R* = .37, *p* = .023; Fig. 5a). Furthermore, in the control group, women scored significantly higher compared to men (W = 102, *p* = .037; Fig. 5b).

## Discussion

The present study on this European population confirmed that the DSD-20 test is suited for olfactory testing. The correlation between DSD-20 and the total score in Sniffin’ Sticks was high (TDI; *R* = .80), and the test correlated with the individual components of the Sniffin’ Sticks test: strongly with odor threshold (*R* = .71) and discrimination (*R* = .73) and moderately with odor identification (*R* = .64). This correspondence between DSD-20 and one of the most common commonly used olfactory tests indicates that the investigated method is an appropriate screening tool for a European population. Furthermore, the correlation coefficient between the DSD-20 test and retest was very high (*R* = .88), particularly when compared to the reliability of other olfactory tests [31]. It is worth mentioning that the coefficient obtained in the present study was even slightly higher than in case of the original method, DSD-40 (*R* = .82; 22).

The major advantages of DSD-20 relate to its simplicity in self-administration, speed of application, portability, and the fact that it can be reused. DSD-20 takes less time than other tests based on odor identification. To complete it, 6–10 min are needed, which makes it particularly beneficial in daily clinical practice, where time is limited. The same applies to the fact that DSD-20 is completely self-administered, both in terms of testing and scoring.

The current study also investigated the influence of demographic factors on test performance among healthy participants, in order to examine its validity. Older participants scored lower in DSD-20 than younger ones, reflecting a typical decline in olfactory performance [32]. Regarding gender, women scored significantly higher than men, confirming previous studies [33]. The cut-off score to diversify between normosmic and hyposmic is the 10th percentile of the distribution of the test scores in a group of young and healthy people, as it has been used in previous studies [34, 35].

Shapiro-Wolf test indicated a non-normal distribution of DSD-20. The scores exhibited a negative, moderate skew and a leptokurtic distribution, with most participants scoring higher on the test. Similar results were reported when evaluating this test on the Brazilian population [29]. Such data distribution has been found before in other olfactory tests [36] that are commonly used in clinical practice. We conclude that DSD-20 can be helpful in many clinical assessments, especially those associated with decreased olfactory performance.

The present study is not free from limitations. Future studies in this field should benefit from including a higher number of participants. However, even with the current sample, the effect size was relatively large, indicating that the quantity of collected data was sufficient. Also, investigating a link between cognitive and affective measurements and DSD-20 would put more light on the possible usage of DSD-20 not only in olfactory testing but also in cognitive screening. Finally, future investigations should also obtain data on how fast participants performed the DSD-20 compared to established tests like the Sniffin’ Sticks or how they enjoyed the test situation.

Overall, the present study confirms previous notions on DSD-20 [37, 38] by demonstrating its high reliability and usefulness in separating patients with hyposmia/anosmia and normosmic controls. Importantly, it also shows, for the first time, the correlation to the Sniffin’ Sticks, all of this in a European population. Based on that, we expect that DSD-20 will become more and more common in routine clinical practice, especially when time and staff are limited.
